# Introgressing Subgenome Components from *Brassica rapa* and *B. carinata* to *B. juncea* for Broadening Its Genetic Base and Exploring Intersubgenomic Heterosis

**DOI:** 10.3389/fpls.2016.01677

**Published:** 2016-11-17

**Authors:** Zili Wei, Meng Wang, Shihao Chang, Chao Wu, Peifa Liu, Jinling Meng, Jun Zou

**Affiliations:** National Key Laboratory of Crop Genetic Improvement, College of Plant Science & Technology, Huazhong Agricultural UniversityWuhan, China

**Keywords:** interspecific hybridization, *Brassica juncea*, subgenomic introgression, heterosis, genetic diversity, novel population, Development of new-type *Brassica juncea* with introgression of subgenomes from related oilseed species.

## Abstract

*Brassica juncea* (A^j^A^j^B^j^B^j^), is an allotetraploid that arose from two diploid species, *B. rapa* (A^r^A^r^) and *B. nigra* (B^n^B^n^). It is an old oilseed crop with unique favorable traits, but the genetic improvement on this species is limited. We developed an approach to broaden its genetic base within several generations by intensive selection. The A^r^ subgenome from the Asian oil crop *B. rapa* (A^r^A^r^) and the B^c^ subgenome from the African oil crop *B. carinata* (B^c^B^c^C^c^C^c^) were combined in a synthesized allohexaploid (A^r^A^r^B^c^B^c^C^c^C^c^), which was crossed with traditional *B. juncea* to generate pentaploid F_1_ hybrids (A^r^A^j^B^c^B^j^C^c^), with subsequent self-pollination to obtain newly synthesized *B. juncea* (A^r/j^A^r^/^j^B^c/j^B^c^/^j^). After intensive cytological screening and phenotypic selection of fertility and agronomic traits, a population of new-type *B. juncea* was obtained and was found to be genetically stable at the F_6_ generation. The new-type *B. juncea* possesses good fertility and rich genetic diversity and is distinctly divergent but not isolated from traditional *B. juncea*, as revealed by population genetic analysis with molecular markers. More than half of its genome was modified, showing exotic introgression and novel variation. In addition to the improvement in some traits of the new-type *B. juncea* lines, a considerable potential for heterosis was observed in inter-subgenomic hybrids between new-type *B. juncea* lines and traditional *B. juncea* accessions. The new-type *B. juncea* exhibited a stable chromosome number and a novel genome composition through multiple generations, providing insight into how to significantly broaden the genetic base of crops with subgenome introgression from their related species and the potential of exploring inter-subgenomic heterosis for hybrid breeding.

## Introduction

*Brassica juncea* (A^j^A^j^B^j^B^j^, *2n* = 36) is a tetraploid that originated from its diploid progenitors, *B. rapa* (A^r^A^r^, *2n* = 20) and *B. nigra* (B^n^B^n^, *2n* = 16). It is deduced that *B. juncea* formed ~0.039−0.055 million years ago, and, based on a recently completed genome sequence, has significantly diverged from its parental species such as frequent gene loss (Yang et al., 2016). Humans have cultivated this species for approximately 6000 years as an oil and vegetable crop (Chen et al., 2013). In some Southern Asian countries such as in India, *B. juncea* is currently a major oilseed crop (Chauhan et al., 2011; Dr. Jayantilal Patel personal communication). Currently, increasing attention is being paid to *B. juncea* worldwide, given its unique and favorable traits, such as resistance to biotic and abiotic stress and a low rate of pod shattering. These traits are desirable in the context of global climate change given its capacity to adapt to heat and other stresses that are commonly encountered under field conditions in Canada, Australia, and many other parts of the world (Woods et al., 1991; Cheung et al., 1997; Burton et al., 2004; Huangfu et al., 2009; Amanullah et al., 2011; Chen et al., 2013). However, genetic improvement on this species is still relatively limited even though previous efforts have significantly increased the yield, particularly increases of seed yield in India (Dr. Zhizheng Chen, personal communication). The low seed yield of *B. juncea* in most of the rapeseed growing area substantially restricts extensive planting of this species worldwide. This is largely due to deleterious changes in its genome from genetic improvement programs and the absence of a novel approach to exploit the benefits associated with heterosis, and an international effort is needed to genetically improve this crop (Pradhan and Pental, 2011). Increasing the seed yield, exploring novel germplasms, and promoting new approaches for hybrid breeding are therefore essential for improving the agricultural production of *B. juncea*.

Alien genomic introgression via interspecific crossing is a common and powerful approach in crop breeding programs: (1) limited introgression of loci of high agronomic value for introducing advantageous genes to produce desired traits, and (2) large-scale random introgressions for broadening the genetic basis of species (Allard, 1960; Zamir, 2001; Bennett et al., 2012; Yu et al., 2012). *Brassica* includes three diploid species (*B. rapa, B. nigra*, and *B. oleracea*) with three basic genomes (A, B, and C) and three tetraploids (*B. juncea, B. napus*, and *B. carinata*) derived from natural crosses between the diploid species, as represented in the “U's triangle” (UN, 1935). Scientists recognized the genomic differentiation of these A, B, and, C genomes in different *Brassica* species and introduced the concept of a “subgenome,” which can be distinguished for each species by using a superscript (or subscript e.g., Chalhoub et al., 2014) of the first letter of the species' name, such as A^j^A^j^B^j^B^j^ for *B. juncea*, A^n^A^n^C^n^C^n^ for *B. napus*, B^c^B^c^C^c^C^c^ for *B. carinata*, A^r^A^r^ for *B. rapa* (Li et al., 2004; Zou et al., 2010, Figure 1). The cytological relationships and genomic homology among the species provide a major opportunity to generate novel *Brassica* germplasms, or even novel species, such as *Brassica* hexaploids, via interspecific hybridization in *Brassica* (Chen et al., 2011; Mason and Batley, 2015). To improve the major rapeseed species, *B. napus*, extensive and successful efforts have been made to broaden the genetic basis, and introduce several specific traits from related species (Prakash and Raut, 1983; Meng et al., 1998; Ren et al., 2000; Xiao et al., 2010; Girke et al., 2012a,b; Li et al., 2014). Utilizing the subgenomic differentiation within and between *Brassica* species would also be helpful to broaden the genetic base of *B. juncea* and explore inter-subgenomic heterosis.

**Figure 1:**
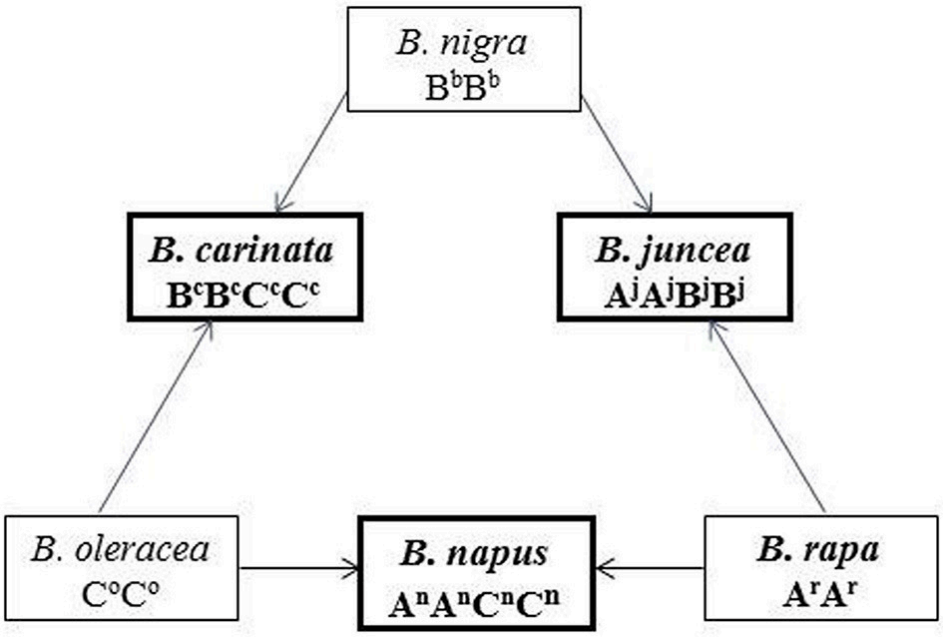
**The U's triangle of ***Brassica*** presenting the cytological relationship of the relation species and their subgenome constitution**. This picture is cited from Zou et al. (2010), which was modified according to UN (1935). The diploid species with the three basic subgenomes (A,B,C) are presented in the three corner of the triangle, and the derived three tetraploid species are presented in the middle of the three lines. Different subgenomes of different species are noted with the superscript of the first letter of each species (Zou et al., 2010).

In a previous study, we obtained allohexaploid *Brassica* (A^r^A^r^B^c^B^c^C^c^C^c^) that was synthesized from the oilseed crops *B. rapa* and *B. carinata* (Tian et al., 2010). The A^r^ subgenome-containing species, *B. rapa*, is a primary oilseed crop in China, with a long cultivation history prior to the import of *B. napus* in the 1950s. *B. rapa* possesses rich genetic diversity and many desirable traits, such as fast growth and tolerance to nutrient-deficient soil and low temperatures (Franks, 2011; Waalen et al., 2011; Ahmed et al., 2012). *B. carinata*, which contains the B genome, is grown in Ethiopia and the area around Northeastern Africa and has unique traits that are desirable in other *Brassica* crops, such as disease resistance, shattering resistance, and drought tolerance (Malik, 1990; Warwick, 2011). Considering the divergent evolution of the A^r^ subgenome and the B^c^ subgenome in different species and in different continents, in the present study, we aimed to broaden the genetic base of the old oilseed crop *B. juncea* by developing lines of new-type *B. juncea* with massive introgression of the A^r^ and B^c^ subgenomes.

## Materials and methods

### Plant materials

Nine allohexaploid lines obtained from crosses between eight accessions of *B. rapa* from China and nine accessions of *B. carinata* from Ethiopia, as shown in Supplementary Table 1 (Tian et al., 2010), were used in this study. A total of 45 accessions of traditional *B. juncea* from various areas of the world were used to perform crosses with the nine hexaploid lines to obtain a new-type of *B. juncea* and to obtain intersubgenomic hybrids. Furthermore, analyses of genetic diversity were performed, in which an additional nine accessions of *B. juncea* were also used (Supplementary Table 2).

### DNA extraction and molecular marker assays

All of the DNA samples (included 82 plants from nine F_2:3_ families, five hexaploid lines and eight accessions of traditional *B. juncea* used as parents of the new-type *B. juncea* lines, six accessions of traditional *B. napus* and two accessions of *B. carinata* of other related species) were extracted from young leaves by a modified version of the DNA extraction method (Doyle, 1990). A total of 208 pairs of fluorescence-labeled primers of simple sequence repeats (SSR, 104 from the A genome, 73 from the C genome and 31 from B genome of *Brassica*) with clear, reproducible and polymorphic amplification products (Supplementary Table 3) were used to evaluate the genetic diversity and population structure of the novel *B. juncea* lines and their parental lines (traditional *B. juncea*) used in this study. The synthesis of the SSR primers, conditions for PCR, and processing of the data generated by capillary sequencing were all described previously (Chen et al., 2010) using an AB3730xl capillary sequencer (Applied Biosystems, USA). An additional set of SSR markers anchored on 31 loci from the B genome in *B. carinata* (Zou et al., 2014) was used to evaluate the introgression of B genomic components (Supplementary Table 3). The markers were scored according to the polymorphism appeared in the parents and population. Those alleles newly appeared or missing in the population compared with the parents were noted as novel allelic variation.

### Cytogenetic observations and GISH and BAC-FISH analyses

The chromosome number in the plants was counted in cells from the wall of the ovary, as described by Tian et al. (2010). For meiotic analysis, young flower buds were fixed in fresh Carnoy's solution for 24 h and then stored in 70% ethanol. Slides of chromosomes from the pollen of mother cells were prepared for GISH (genomic *in situ* hybridization) and BAC-FISH (bacterial artificial chromosome fluorescence *in situ* hybridization) based on the procedures of Ge and Li (2007). Genomic DNA from *B. nigra* cv. Giebra (labeled with digoxigenin-11-dUTP; Roche, Basel, Switzerland) and plasmid DNA from BAC BoB014O06 (labeled with biotin-11-dUTP; provided by Susan J. Armstrong, University of Birmingham, Birmingham, UK, the probe distribute on all C chromosomes) were used to identify B- and C-genome chromosomes using GISH and BAC-FISH analysis. Probe preparation and *in situ* hybridization were performed using the methods described by Cui et al. (2012).

### Field observation of agronomic traits and evaluation of heterosis potential

Plants were mainly grown in Wuhan (located in central China), but some plants in the F_4_ generation were also grown in Hezheng (northwest China). The proportion of seed sets for each of the new-type *B. juncea* line and cultivars of traditional *B. juncea* was accounted for according to the average seed number of 10-30 siliques evaluated with 4-6 plants each line. A total of 445 F_4_ plants from 72 F_3:4_ families of new-type *B. juncea*, and a total of 81 F_6_ plants from 20 F_5:6_ families of new-type *B. juncea* were measured. The kilo-seed weight of each line was evaluated with the average weight of 1000 seeds from each of the investigated plants.

Inter-subgenomic hybrids between the lines of new-type *B. juncea* in early generations (F_3_, F_4_, and F_5_) and traditional *B. juncea* were generated by hand crossing and planted along with their parents. The hybrids derived from the F_3_ lines were grown in Hezheng as a spring-type crop in 2012 and in Wuhan as a semi-winter-type crop from 2012 to 2013 to evaluate heterosis potential for seed yield. All plants were grown in two rows, with each row 1.8 m in length and 0.25 m between rows. The hybrids derived from the F_4_ lines and F_5_ lines were grown in Wuhan from 2013 to 2014, and 2014 to 2015, respectively, with two replicates (24 plants with 0.3 m space among plants for each replication) and three replicates (21 plants with 0.3 m among plants for each replication), respectively. The middle parent heterosis and superior parent heterosis of the hybrids were calculated. Individual significant differences and overall significant differences among hybrids and their parents across multiple combinations in each year was evaluated by the F test at the significance level of *P* < 0.05, using the multiple comparison model under a single factor with repeats and the multiple comparison model under double factors with repeats in SAS 8.1 (SAS Institute, 1999), respectively.

### Population genetic analysis

The genetic diversity and genetic distance between different plants was evaluated with molecular markers analyzed using Primer 6 software (Clarke and Gorley, 2006). The Shannon index was calculated using POPGENE 1.31 (Yeh et al., 1999). The phylogenetic tree was analyzed based on Nei's genetic distance (Nei et al., 1983) using the clustering method, employing a neighbor-joining tree with 1000 permutations, using POPTREE 2 (Takezaki et al., 2010). After eliminating improper markers with allelic frequencies less than 10 or >10% missing data, the population structure was analyzed by Bayesian clustering and the “admixture model” with STRUCTURE 2.2 (Pritchard et al., 2000; Falush et al., 2003). The length of burn-in time and replication number were both set to 100,000 in each run. To identify and determine the most probable population number (K), we calculated the ΔK values of K from 1 to 10 replicate runs for each K, and selected the K value corresponding to the peak of the ΔK graph after plotting (Evanno et al., 2005).

## Results

### The strategy and process of creating the new-type *B. juncea*

**Figure 2:**
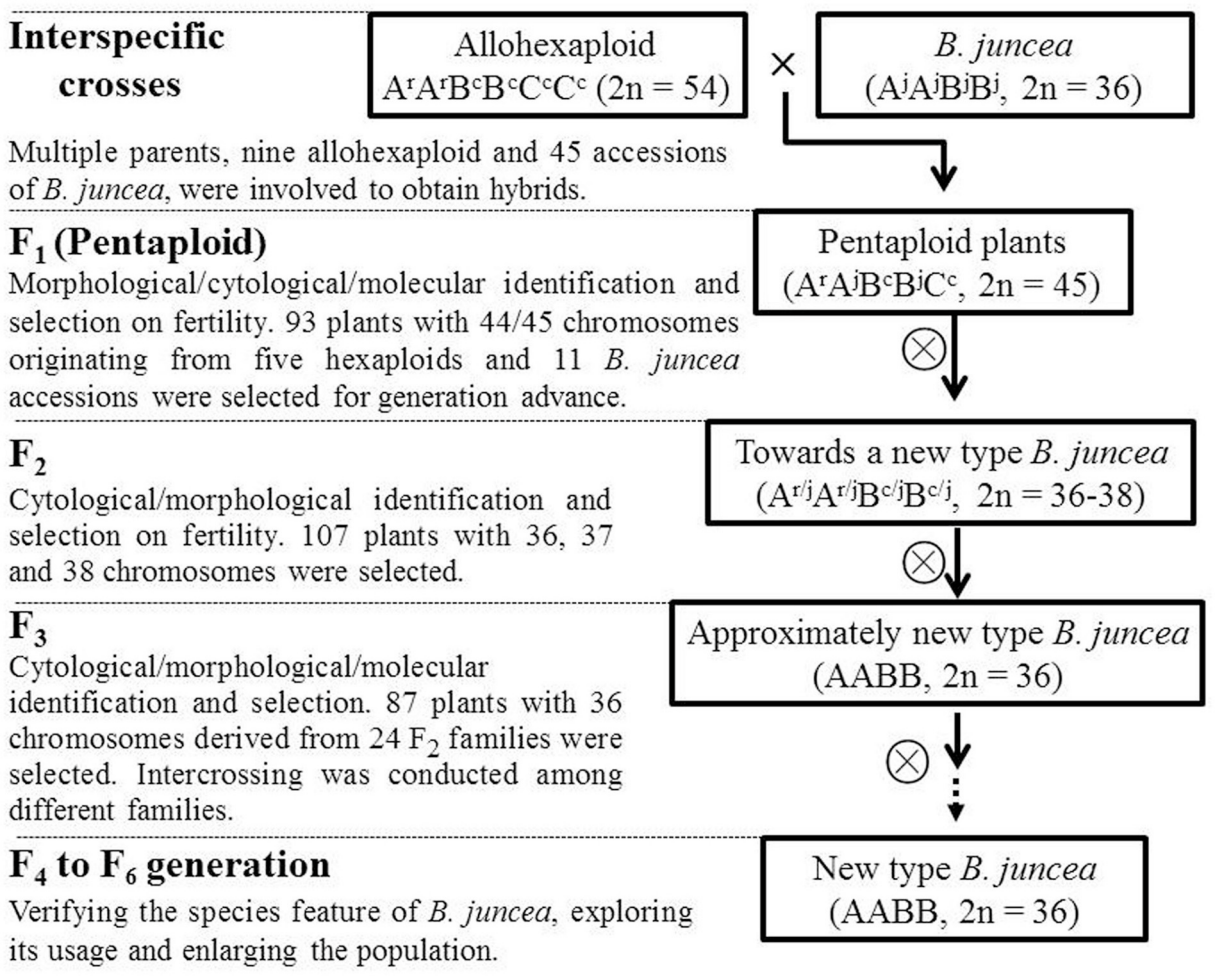
**Strategy used to generate a population of new-type ***B. juncea*****. The *Brassica* allohexaploid in which the A^r^ and B^c^ subgenomes from *B. carinata* (B^c^B^c^C^c^C^c^) and *B. rapa* (A^r^A^r^) (Tian et al., 2010) were combined was used as the donor of exotic subgenomes. The first step in the current study involved interspecific crosses between various hexaploid and traditional *B. juncea* to transfer the A^r^ and B^c^ subgenomes to *B. juncea*. Successive phenotypic and cytological selection was then conducted up to the F_6_ generation, whereas genomic surveying was mainly conducted at the F_3_ generation.

A set of *Brassica* allohexaploids in which the A^r^ and B^c^ subgenomes of *B. carinata* (B^c^B^c^C^c^C^c^) and *B. rapa* (A^r^A^r^) (Tian et al., 2010) were combined was used as donors for the introgression of exotic subgenomes into the traditional *B. juncea* genome. Subsequently, morphological/molecular/cytological identification and intensive selection for generations were performed (Figure 2).

Initially, nine hexaploid lines were successfully crossed with 45 accessions of traditional *B. juncea*, which generated thousands of hybrid seeds (approximately 15,000 seeds) derived from 108 combinations. The cross success rate for different pentaploid combinations varied from 0.1 to 1, with an average cross success rate value of 0.8 and 5.7 seeds per silique. The seeds per silique in the hexaploid seeds and traditional *B. juncea* parents varied from 0.9 to 3.7 and 9.6 to 16.7, respectively. The authenticity of the hybrids was verified using DNA markers after initial identification by morphological observation (Supplementary Figure 1), and the chromosome number of each hybrid was counted. After obtaining pentaploid progeny, self-pollination was performed across generations.

The seeds from 93 identified true hybrids gave rise to the F_2_ generation. The F_2_ generation was screened for plants with the correct number (or close to the correct number) of chromosomes (36) as well as good fertility (defined as an abundance of pollen in the flowers and more than eight seeds per silique). The 36 chromosomes were expected to consist of the total 2 A and 2 B genomes, without any of the C genome chromosome. When the genome of A, B and C was integrated in hexaploids, the C genome was more easily lost because the genome stability was B>A>C (Zhou et al., 2016). The pedigrees of the introgressed lines were established according to the original interspecific crosses for the pentaploids. Then, selfing of each pentaploid resulted in different F_2_ families. Subsequently, the other round of phenotypic selection was performed at the F_3_ generation with a focus on families descended from plants with 36 chromosomes. A genomic analysis was performed to evaluate whether the F_3_ plants diverged from the traditional *B. juncea* lines due to the massive exotic introgression. In the F_4_ generation, GISH and FISH analyses were used to confirm the correct genomic constitution of the lines of new-type *B. juncea* (AABB). Chromosomes were further assessed using the lines at the F_6_ generation.

### The consequence of cytogenetic selection on plants with the target number of chromosomes

**Figure 3:**
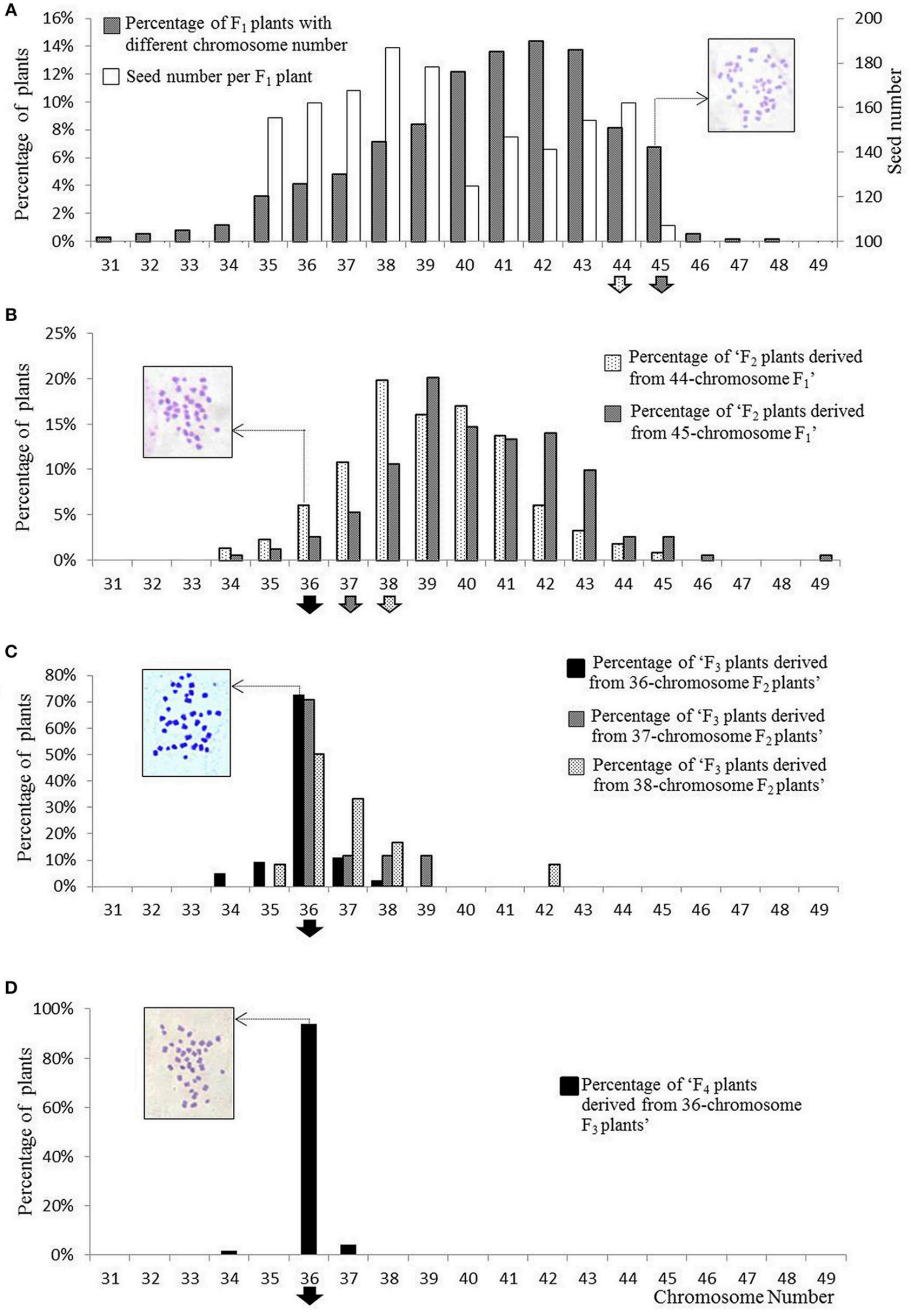
**The distribution of plants with different chromosome numbers at four successive segregating generations, from pentaploid F_**1**_ to F_**4**_. (A)** The segregation of F_1_ plants with different chromosome numbers and seed set. In the F_1_ generation, the number of seeds produced from each category of plants with a different chromosome number is also presented. Self-pollinated seeds from F_1_ plants with 44 or 45 chromosomes were selected (as indicated by the arrows on the bottom) to produce offspring. **(B)** The segregation of plants with different chromosome numbers at the F_2_ generation, which originated separately from F_1_ plants with 44 or 45 chromosomes. Plants with 36 to 38 chromosomes were selected to produce offspring. **(C)** The segregation of the plants with different chromosome number in the F_3_ generation, 50 to 80% of plants derived from F_2_ plants with 38, 37, or 36 chromosomes were identified as having 36 chromosomes; only plants with 36 chromosomes were selected as new-type *B. juncea*. **(D)** The majority of the plants in the F_4_ generation inherited 36 chromosomes. The arrows indicating the selected plants were filled with colors and grains that were the same as the columns in the picture in the next generation. The cytological photographs illustrate the correct chromosome numbers of 45, 36, 36, and 36 in the pentaploids, target F_2_ plants, and new-type *B. juncea* (F_3_ and F_4_), respectively.

Because meiosis in the artificially synthesized hexaploids cannot be completely normal (Tian et al., 2010), the F_1_ hybrids derived from the 108 interspecific crosses between the hexaploid and tetraploid *B. juncea* might have a segregated chromosome composition deviate from the expected 45 chromosomes. After removing the abnormal seedlings and maternal plants, more than 700 F_1_ plants bearing self-pollinated seeds were obtained and subjected to chromosome counting. Only 6.73% of the plants had the expected number of chromosomes (45) for pentaploids (Figure 3A). To avoid incorrectly discarding plants with the appropriate number of chromosomes, the plants scored with 44 chromosomes were also regarded as potential pentaploid. Consequently, self-pollinated seeds harvested from 93 F_1_ plants assumed to be pentaploid, were selected to produce the F_2_ generation.

Approximately 1000 F_2_ plants were obtained, and approximately one-third of the plants exhibited abundant pollen (as observed by eye); they were selected as candidate tetraploid plants for chromosomal counting. In the F_2_ population descended from the F_1_ specimens with 45 chromosomes, only 2.69% of the plants exhibited 36 chromosomes; most of the plants had extra chromosomes (Figure 3B). To obtain more euploid progeny (A^r/j^A^r/j^B^c/j^B^c/j^, *2n* = 36) in the next generation, F_2_ plants with 37 or 38 chromosomes (which may be aneuploid with one or two extra chromosomes that could be lost in the next cycle of meiosis) were selected together with plants with 36 chromosomes to produce the F_3_ generation via self-pollination.

More than 2000 F_3_ plants were obtained from various 24 F_2_ plants derived from different pentaploids with 36, 37, or 38 chromosomes, which were established as 24 derived F_2:3_ families according to the origin of the pentaploid cross in the F_2_ generation. The chromosome numbers were assessed for those fertile plants that generated abundant pollen at flowering and produced a large number of seeds. Of the fertile plants, 70% (119 of 170) were regarded as euploid (*2n* = 36), 85% of the inferred euploid specimens were derived from 13 F_2_ plants with 36 chromosomes (Table 1, Figure 3C), and 5 of the 13 F_2_ families constituted 69% of the inferred F_3_ euploid plants. The plants derived from 37- or 38-chromosome F_2_ plants accounted for 15% of the inferred euploid specimens in the F_3_ generation (Supplementary Tables 4, 5).

**Table 1:** **The number of plants and families investigated and identified in the F_3_ generation**.

**Chromosome number in the F_2_ generation**	**Total F_3_ plants/ F_2:3_ families grown to maturity**	**No. of F_3_ plants/ showing plentiful pollen**	No. of fertile F_3_ plants^*^/ F_2:3_ families checked	**No. of F_3_ plants/ F_2:3_ families with 36 chromosomes**
36	1200/17	465/14	139/13	101/13
37	450/23	344/21	17/9	12/7
38	550/37	276/29	14/9	6/4
Total	2200/77	1085/64	170/31	119/24

* *A plant with ≥ 8 seeds/silique was regarded as a fertile plant*.

**Figure 4:**
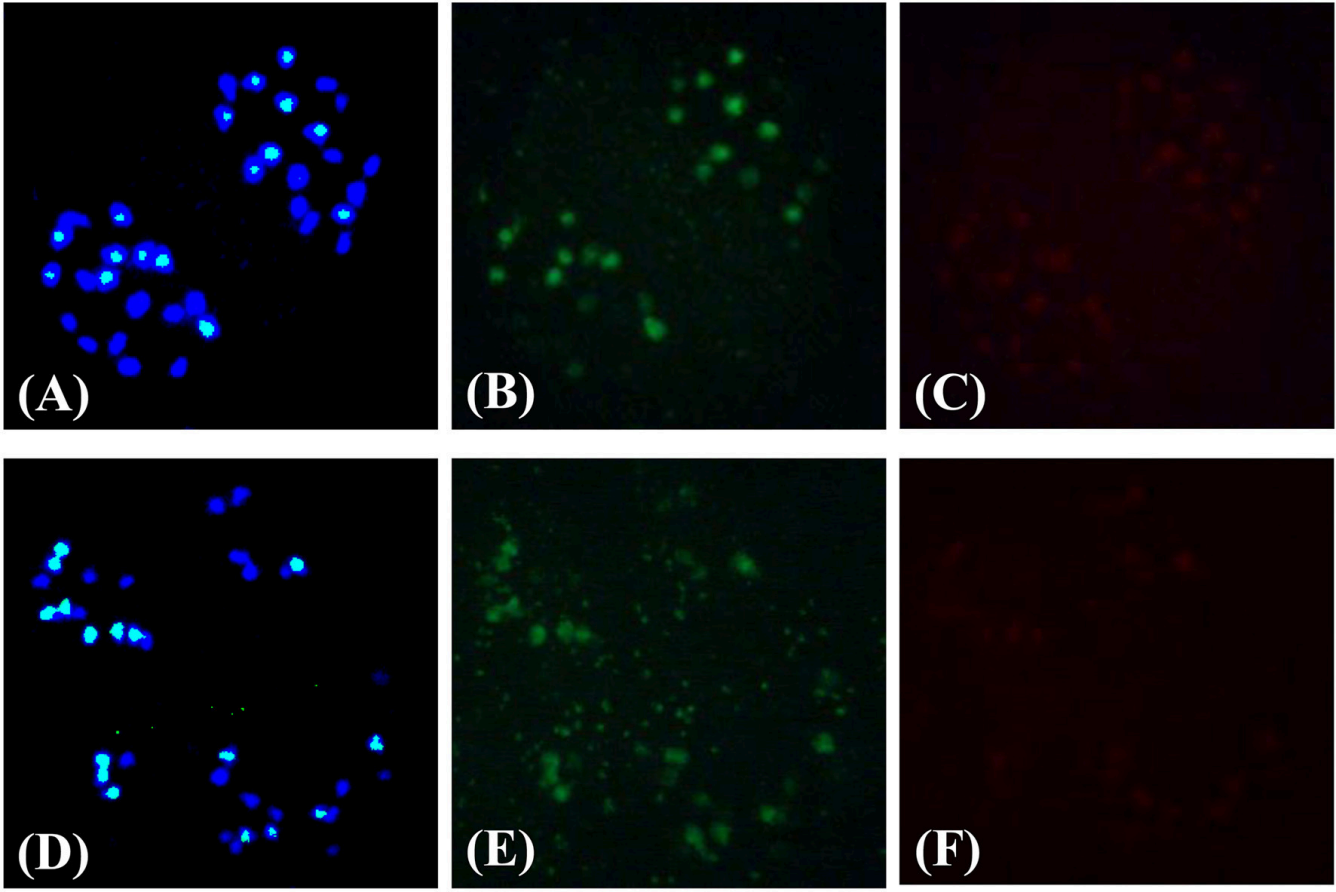
**Demonstration of the chromosome composition of new-type ***B. juncea*** lines via cytological observations**. A pollen mother cell in meiotic anaphase I depicting the chromosome composition of a new-type *B. juncea* plant, C18J23-1-2-8, at the F_4_ generation **(A–C)** compared with traditional *B. juncea* accession, J56 **(D–F)**, as revealed by *in situ* hybridization. **(A,D)** Merged images of DAPI staining (blue) and a B genome DNA probe (bluish green). Sixteen chromosomes from the B genome (bluish green) were distinguished from 36 chromosomes in total, and separated equally into two nuclei. Twenty chromosomes from the A genome (pure blue) were observed. **(B,E)** Images of a B-genome probe (which appears green without DAPI interference); only 16 chromosomes from the B genome were distinguished. **(C,F)** Images of a C-genome probe (red); no obvious red signals were found, suggesting that the 20 chromosomes in **(A,D)** with a blue color were from the A genome and not the C genome.

Approximately 2500 F_4_ plants derived from 87 F_3_ plants with 36 chromosomes were obtained. Overall, 94% of the plants, counted from 116 F_4_ individuals, were euploid (*2n* = 36) (Figure 3D, Supplementary Table 6). To verify that the “inferred euploid” specimens with 36 chromosomes exhibited AABB euploidy, the chromosome composition of five F_4_ plants with 36 chromosomes was investigated by cytological observation. All of the plants exhibited normal behavior at meiosis in pollen mother cells with 20 chromosomes from the A genome and 16 chromosomes from the B genome (Figure 4). We further counted the chromosome numbers of 78 F_6_ plants with normal fertility derived from 21 F_4_ families and found that 96.2% of the F_6_ plants had 36 chromosomes (Supplementary Tables 6, 7), and all of the plants descended from seven 36-chromosome F_3_/F_4_ families (25 of the 78 plants) consistently exhibited 36 chromosomes as well. It indicated that the lines of new-type *B. juncea* were genetically stable, at least, from the F_6_ generation.

### The consequences of selection on plant fertility

**Figure 5:**
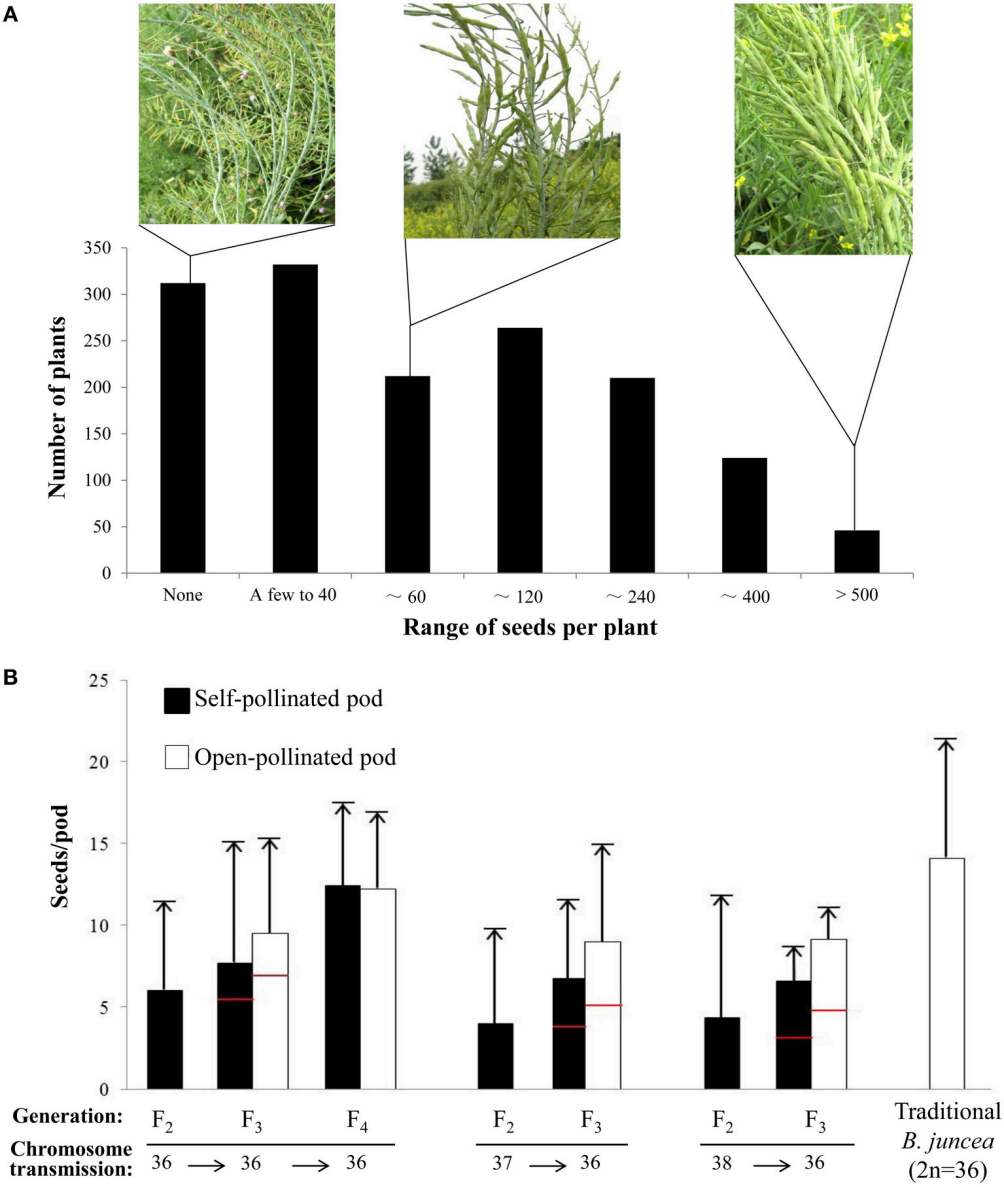
**Plant fertility in different generations after interspecific crossing**. **(A)** The categories of plants exhibiting different fertility and levels of seed set in the F_1_ generation. The levels of seed set varied markedly from zero to hundreds of seeds produced per plant. **(B)** The seed set rate (seeds/silique) was determined in the F_2_, F_3_, and F_4_ generations by an analysis of 90, 55, and 72 plants, respectively. The horizontal arrows at the bottom indicate the consequences of chromosome transmission from generation to generation, and the vertical arrows above the columns indicate the maximal number of seeds per silique in one plant. The red bars in the column of the F_3_ generation indicate the mean value of seeds per silique counted from 675 plants; chromosomes were not counted in most of these plants. Six cultivars of traditional *B. juncea* were used as a control.

The seed set of plants in the F_1_ generation varied widely among individually bagged plants, ranging from no seed set to close to a normal level. One-fifth of the F_1_ plants were seedless, while approximately half of the plants yielded more than 100 self-pollinated seeds, and a small proportion of plants (approximately 4%) produced more than 500 seeds (Figure 5A).

Plants with 36, 37, and 38 chromosomes were used to investigate the fertility of the F_2_ generation. The plants with 36 chromosomes exhibited the highest rate of seed set (6.04 seeds/silique), which was significantly higher (*P* < 0.05) than the seed set rate of plants with 37 or 38 chromosomes (Supplementary Table 5). Some of the 36-chromosome F_2_ plants borne more than 10 seeds per silique (Figure 5B).

The majority of F_3_ plants exhibited good fertility and flowered normally with abundant pollen (Table 1, Supplementary Figure 2). However, the F_3_ progeny derived from the 36-chromosome F_2_ plants had higher rates of seed set than the offspring derived from 37- or 38-chromosome F_2_ plants, as judged by the number of seeds per silique and the seed yield per plant (Figure 5B, Supplementary Table 8). Compared with the F_2_ generation, the number of seeds per silique in the plants with 36 chromosomes increased in the F_3_ generation regardless of whether they were derived from 36-, 37-, or 38-chromosome F_2_ plants.

The number of seeds per silique increased again in the F_4_ generation, and the best F_3_-derived lines with 36 chromosomes had a good seed set rate that was comparable to that of traditional *B. juncea* lines (Table 2). The results of cross-pollination with traditional *B. juncea* accessions showed that the lines of new-type *B. juncea* were not sexually isolated from but were instead compatible with traditional cultivars of *B. Juncea* (Supplementary Figure 2).

**Table 2:** **Extent of trait variation in the ‘new-type ***B. juncea*** lines’ (F_**4**_-F_**6**_) and traditional accessions of ***B. juncea*****.

Agronomic trait^*^	**New-type *B. juncea* lines**			**Traditional *B. juncea* accessions**	
	**Generation**	**Mean ±*SD***	**Range**	**Mean ±*SD***	**Range**
Plant height (cm)		179.04±18.90	132~232	154.10±24.95	101.71~187.00
Branch number		9.47±3.01	5.75~18.50	8.76±1.98	6.08~11.10
Silique number per plant	F4^*^	400±156.40	172.89~948.51	401.32±177.91	173.67~767.53
Kilo-seed weight (g)		2.01±0.39	1.35~3.40	1.71±0.46	1.22~2.48
Seed number (per silique)		11.46±1.96	7.69~15.54	12.41±2.09	9.61~16.72
Seed yield (g/plant)		9.34±3.42	3.31~22.80	7.52±2.55	2.13~7.79
Theoretical seed yield (g/plant)^**^		9.21		8.52	
Kilo-seed weight (g)	F6^***^	2.00±0.37	1.38~2.57	1.88±0.42	1.27~2.64
Seed number (per silique)		12.81±2.70	8.39~20.34	12.40±1.43	8.98~13.98

* *Data were calculated from 445 F_4_ plants from 72 F_3:4_ families of new-type B. juncea and compared with the accessions of traditional B. juncea*.

** *Calculated from Silique number × Kilo-seed weight × Seed number/1,000. The kilo-seed weight is the weight of 1000 seeds, therefore, we divided 1000 to calculate the seed yield per plant*.

*** *Data were calculated from 81 F_6_ plants from 20 F_5:6_ families of new-type B. juncea compared with ten accessions of traditional B. juncea*.

### Genetic diversity in a population of new-type *B. juncea* compared with traditional *B. juncea* accessions

**Figure 6:**
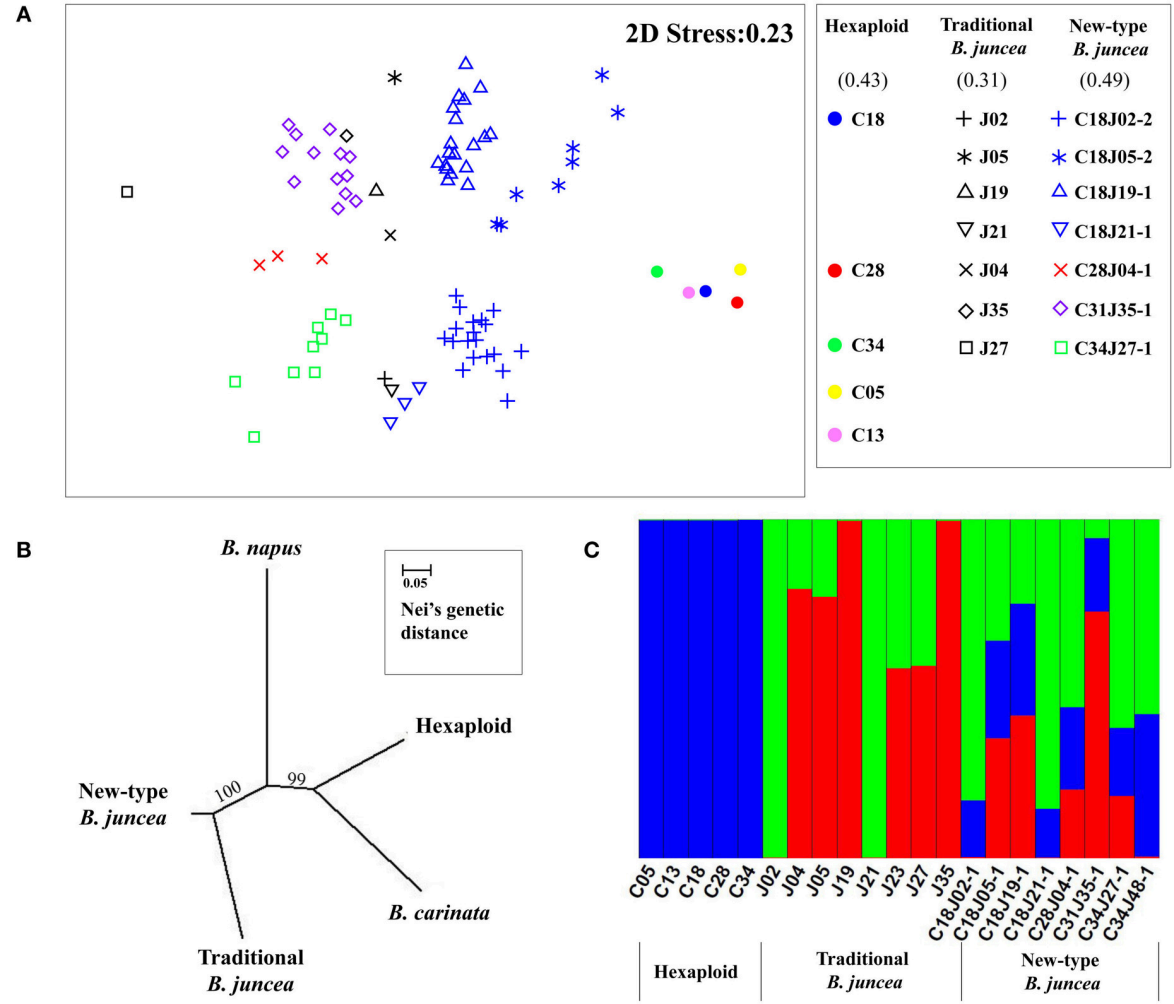
**Genetic divergence between the new-type ***B. juncea*** lines and relative species**. **(A)** The genetic differences between the new-type *B. juncea* lines in the F_3_ generation and their parents were revealed by principle ordination analysis with multi-dimensional scaling. We draw three ovals to outline the three genetic clusters of the new-type *B. juncea*, traditional *B. juncea* and hexaploid, respectively. The logos for different lines of new-type *B. juncea* were designed to have the same color as their hexaploid parents (shown in cycles with different colors) and the same shape as their *B. juncea* parents (shown with different geometric shapes). The arrows at the upper right indicate two plants from F_2:3_ family C18J0502 with remarkable genetic distance from traditional *B. juncea*. The key to the symbols is shown on the right, and the Shannon Index for the new-type *B. juncea* and their parental species is shown in the brackets. **(B)** Phylogenetic tree of eight families of new-type *B. juncea*, eight accessions of traditional *B. juncea*, two accessions of *B. carinata*, five hexaploid lines and two accessions of *B. napus*. The new-type *B. juncea* formed a group that could be distinguished from traditional *B. juncea* and other species, including *B. napus* (AACC), another tetraploid species in the genus *Brassica*. The length of the branches represents Nei's genetic distance, and the numerals above the branches indicate the reliability by bootstrap analysis. **(C)** The population structure of new-type *B. juncea* and its parental species at *K* = 3 (Supplementary Figure 3). The blue color represents the genetic structure of hexaploids, while the red and green color represent the genetic structure of traditional *B. juncea*. The genetic structure of new-type *B. juncea* reflected the membership fractions from both parental species (hexaploid and traditional *B. juncea*).

A set of 978 SSR markers was used to analyze the genetic diversity of the new-type *B. juncea* lines compared with their parental species. Of them, 44.2, 17.5, and 9.6% markers could be assigned to A, B, C genome according to the origin of the alleles amplified in different parental species, respectively. However, 28.7% of the markers could not be distinguished because of multiple amplified products in different genomes. For the plants from the same family, we estimated the percentage of the introgression according to the origin of the alleles from both parents (Supplementary Table 9). Those alleles were not originated from both of the parents, but newly appeared or disappeared in the new-type *B. juncea* lines were noted as novel allelic variation. Averagely 39.2, 25.5, and 35.4% of the genome of new-type *B. juncea* were constituted with the introgression of the traditional *B. juncea* parents, hexaploid parents and novel allelic variation (Supplementary Table 9). For the introgression from hexaploid parents, 55.5 and 15.1% of the alleles were originated from the A and B genome, respectively, but 29.4% of them could not be assigned. The new-type *B. juncea* lines exhibited substantial genetic distance from their hexaploid parents, as assayed via principal ordination analysis (Figure 6A). The F_2:3_ family, C34J27-1, along with most of other F_2:3_ families, exhibited clear genetic differences relative to their tetraploid parents and traditional *B. juncea* accessions. Most of the F_2:3_ families of new-type *B. juncea*, especially C18J05-2, also exhibited marked polymorphisms within their families and demonstrated increased genetic variation as a whole compared with traditional *B. juncea* accessions. An estimate of the gene diversity index between the different populations indicated that the new-type *B. juncea* lines exhibited the richest genetic diversity and the highest Shannon Index (0.49), which was considerably higher than that of traditional *B. juncea* accessions (0.31). A phylogenetic tree revealed that the new-type *B. juncea* was obviously separated from traditional *B. juncea* and had a greater genetic distance from other *Brassica* species than traditional *B. juncea* (Figure 6B). Population genetic structure analyses indicated that three apparent genetic clusters existed among the investigated lines and their parents (Figure 6C, Supplementary Figure 3). The new-type *B. juncea* lines inherited major genetic components from both parents (the hexaploids and traditional *B. juncea*).

### Potential for trait variation and inter-subgenomic heterosis in the early generations of the new-type *B. juncea* lines

In total, 119 of the best F_3_ plants with marked variation in morphology and agronomic traits were selected from 24 F_2:3_ families, which represent 14 combinations involving five hexaploid lines (each synthesized from different accessions of *B. carinata* and *B. rapa*) and 11 accessions of traditional *B. juncea* (Supplementary Table 10). After testing the seed yield and yield components, seeds from 76 F_3_ plants representing all of the founder parents and cross combinations were ultimately sown in the field to generate the F_4_ generation, which resulted in approximately 2500 plants that exhibited distinct differences from their parental species and presented comparable agronomic traits to traditional *B. juncea* (Table 2, Supplementary Figures 4A–D). The agronomic and morphological traits of the new-type *B. juncea* lines were further improved at the F_6_ generation through selection, resulting in an obviously increased seed number per pod.

**Figure 7:**
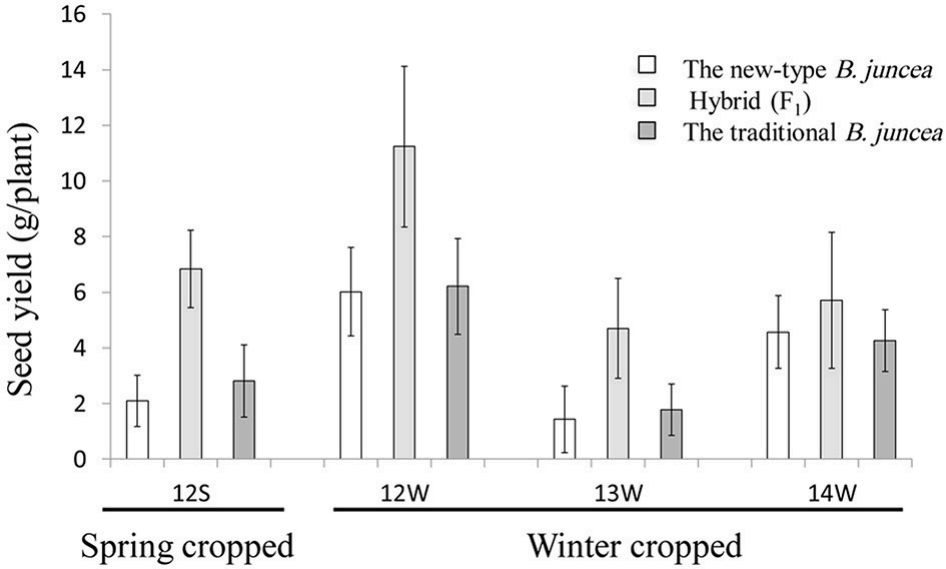
**Seed yield of new-type ***B. juncea*** lines, traditional ***B. juncea*** accessions and the hybrids between them**. The spring crop data were collected from nine F_3_ lines of new-type *B. juncea*, eight cultivars of traditional *B. juncea*, and six hybrids between the new-type *B. juncea* lines and traditional *B. juncea* accessions at the spring-type growing environment in 2012 (environment abbreviation as 12S), and the winter crop data were collected from 10 F_3_ lines of new-type *B. juncea*, five cultivars of traditional *B. juncea*, and 10 hybrids in 2012–2013 at the winter-type environment (environment abbreviation as 12W). The winter crop data for 2013–2014 and 2014–2015 in the winter-type environment (environment abbreviation as 13 and 14W) were successively collected from eight F_4_ and 10 F_5_ lines of new-type *B. juncea*, five and seven cultivars of traditional *B. juncea*, and 13 and 21 derived hybrids, respectively (for more detail, see Supplementary Table 10).

We estimated the heterosis potential for the seed yield of the inter-subgenomic hybrids between the new-type *B. juncea* lines in different early generations (F_3_, F_4_, and F_5_) and traditional *B. juncea* accessions. The seed yield of the hybrids was significantly increased compared with parental accessions when grown as a spring crop in the northwest of China and as winter crop rapeseeds in central China (Figure 7, Supplementary Table 11 and Supplementary Figure 4E). The average over superior parent heteoris and mid-parent heterosis of the tested hybrids across environments were 60 and 96%, respectively. However, the heterosis potential of different lines also varied extensively.

## Discussion

To broaden the genetic base of traditional *B. juncea*, we demonstrated an approach to generate new-type *B. juncea* lines with introgression of subgenomes from its related oilseed species, *B. rapa* and *B. carinata*, via interspecific crossing and intensive selection. We performed direct selection on the hybrid offspring obtained from interspecific crosses between artificial allohexaploid (A^r^A^r^B^c^B^c^C^c^C^c^) lines and traditional *B. juncea* (A^j^A^j^B^j^B^j^) accessions. Subsequently, we conducted intensive phenotypic selection and cytological screening in the offspring of the pentaploids under self-pollination over three successive generations (Figure 2). As a result, a population of new-type *B. juncea* founded with multiple parents was developed (Figures 4–6). The new-type *B. juncea* was stable in terms of the chromosome composition up to the F_6_ generation (Supplementary Tables 6, 7), presented rich genetic diversity within the population, and diverged from, but was not isolated from traditional *B. juncea* (Figure 6). The traits of the new-type *B. juncea* lines, were comparable to the traditional parental *B. juncea* (Table 2, Supplementary Figure 4). Strong seed yield heterosis was observed in the hybrids between new-type *B. juncea* and traditional *B. juncea* accessions from different environments (Figure 7, Supplementary Table 11). The novel germplasm may be a valuable resource to bring the old *Brassica* oilseed crop into the world of modern breeding, especially due to its unique favorable traits for global climate changing such as drought-resistance. This work helps to elucidate how plant crops may receive genome-wide benefits when bred with related species.

Our strategy also demonstrated the efficiency of direct selection for introducing considerable exotic genomic components to the donor species. The analysis of genetic structure showed that each of the investigated lines of new-type *B. juncea* exhibited a large membership fraction from the hexaploid parents synthesized from *B. carinata* and *B. rapa*. One-third of the total estimated alleles, according to estimation by a total of 978 molecular markers (Supplementary Table 9), showed novel variation in the new-type *B. juncea* lines compared with the parents, including newly appearing alleles and missing alleles compared with the parents. Novel allelic variation has been frequently observed in the progeny of interspecific hybrids in plants (Pontes et al., 2004; Han et al., 2005; Wang et al., 2010; Zou et al., 2011). The appearance of novel alleles, including new alleles and missing alleles, suggests extensive genome reshuffling and recombination between the three *Brassica* genomes in these lines. The genome was altered by thousands of genes via exotic introgression and induced novel variation. Therefore, it is not surprising that the new-type *B. juncea* diverges from traditional *B. juncea* as well as relative species, as revealed by population genetic analysis of a phylogenetic tree and assessment of genetic diversity through principal ordination analysis (Figure 6). Genetic diversity was achieved by using multiple founder parents (five different hexaploids and 11 cultivars of traditional *B. juncea*) and maintaining a relatively large population, as more than 100 plants were selected from thousands of plants grown in each generation, in contrast to a few offspring obtained from a few combinations that are routinely used for addressing the problems caused by reproductive barriers in distant crosses (Kovach and McCouch, 2008; Jena, 2010; Camadro et al., 2012). Further studies to analyze the novel genomic variation of the new-type *B. juncea* lines would provide more information and insights to understand the genomic changes.

To create a novel germplasm for a given species, a balance should be sought between genetic diversity, fertility and chromosome stability. Considering that the gametes originating from the hexaploid parents would be abnormal (Plummer et al., 2010; Tian et al., 2010; Chen et al., 2011) and that errors may occur when counting a large number of chromosomes in each cell, we adopted F_1_ plants with 44 to 45 chromosomes as candidates during the process of screening for pentaploids (*2n* = 45). Actually, approximately two-thirds of the plants that met our selection criteria on chromosome number (*2n* = 36) were derived from “pseudo-pentaploid” specimens with 44 chromosomes. But we also selected F_2_ plants with 37 and 38 chromosomes (15% of the plants with features of interest to the new-type *B. juncea*) and only retained those plants with 36 chromosomes at the F_3_ generation. At F_3_ generation, we firstly checked the pollen amount, then investigated the seed set of the plants with good pollen. After selection on the pollen amount and seed set, we performed chromosome counting for the plants with good pollen and seed set, and selected those plants with 36 chromosomes to next generation. A significantly increased percentage of plants with a stable chromosome number (*2n* = 36) was observed from the F_2_ generation to F_4_ generation. This implies that the new-type *B. juncea* was chromosomally stable after intensive selection in a few generations (Supplementary Table 4). Selecting relatively fertile plants to assess chromosome number increased the efficiency of chromosome counting and also improved the most important agronomic trait for the new-type *B. juncea* population. This strategy might provide a reference for improving the efficiency of generating novel *Brassica* synthetics.

In early generations, the new-type *B. juncea* lines presented comparable agronomic traits to traditional *B. juncea* accessions (Table 2). These traits could be further improved via recurrent selection, which has been demonstrated to be an efficient means of population improvement in crops (Hallauer and Carena, 2012; de Morais et al., 2015; Shelton and Tracy, 2015). Additionally, favorable traits from two different groups of traditional *B. juncea* (Pradhan et al., 1993; Chen et al., 2013) and exotic genome components from *B. napus* (A^n^A^n^C^n^C^n^) (which has been intensively improved by modern breeding and is most widely grown as an oilseed *Brassica* crop) can be introgressed into the pool via recurrent selection. The latter strategy has been demonstrated as a good approach by a group of Indian scientists who developed lines of new-type *B. juncea* with an A^n^A^n^B^c^B^c^ constitution from octoploid A^n^A^n^B^c^B^c^C^c^C^c^C^n^C^n^ plants derived from interspecific crosses between *B. napus* (A^n^A^n^C^n^C^n^) and *B. carinata* (Gupta et al., 2015).

The size of the B genome of *B. nigra* has been reported to be either 632 Mb (larger than that of *B. rapa*, at 529 Mb; Johnston et al., 2005) or close to that of *B. rapa* (Navabi et al., 2013). The results of a genome sequencing project revealed that there are 41,174 protein-coding genes in the A^r^ genome of *B. rapa* (Wang et al., 2011); therefore, it can be estimated that *B. juncea* harboring the AB genome can exhibit approximately 80,000 genes. It was estimated that a considerable percentage (average 25.5% per line and 31.3 % in the total population) of A^r^/B^c^ components were introgressed into the genome of the new-type *B. juncea* (Supplementary Table 9), corresponding to approximately 20,400 genes per line and 25,040 genes in the total population. This massive introgression of alien genes, including numerous favorable alleles, would comprehensively alter the old genome of *B. juncea* and broaden the genetic base of this species. However, abundant space remains for thousands of alien genes to be further introduced in the genome of new-type *B. juncea*.

An obvious heterosis potential for seed yield is observed in the inter-subgenomic hybrids between the new-type *B. juncea* lines and traditional *B. juncea* accessions (Figure 7), which can be explained by the clear genetic distance between the new-type *B. juncea* and traditional *B. juncea*. Strong heterosis was also observed for other traits, such as seed weight (Supplementary Figure 4E). Further estimates of the trait variation and heterosis potential of the new-type *B. juncea* might be evaluated more precisely based on field traits, assessed in large blocks and multiple locations in late generations. The potential for inter-subgenomic heterosis would be strengthened by pyramiding higher introgression, by intercrossing new-type *B. juncea* plants and selection based on genomic components and agronomic traits, as shown in the new-type *B. napus* (Zou et al., 2010). However, in further estimation of the hybrid heterosis for seed yield, the possibility of introducing a male-sterile system into the population of new-type *B. juncea* should be considered.

In modern breeding systems, more importance is given to the sustainability of plant breeding, which urgently requires a high level of genetic diversity among breeding lines and elite cultivars with resource-conserving and environmentally friendly characteristics (Falk, 2010). This study may provide insights into the creation of novel germplasm pools for crops by exploring the genetic resources from related species, especially species in which subgenomes or subspecies already exist, such as wheat, rice, cotton and oil palm. We anticipate that with the recent rapid development of genomic technologies, such as high-throughput genotyping, genome sequencing, genome-wide association studies, and genomic selection, it has become possible for novel germplasms with massive exotic introgression to be appropriately evaluated and effectively used in crop breeding (Mahmood et al., 2007; Navabi et al., 2011; Nakaya and Isobe, 2012).

## Author contributions

ZW and JZ performed the research, analyzed the data and wrote the manuscript. MW and SC performed field experiments and cytological observations. MW and CW screened the molecular markers. PL analyzed the molecular marker data. JZ and JM designed the research, revised the manuscript, and funded the project. All authors read and approved the final manuscript.

### Conflict of interest statement

The authors declare that the research was conducted in the absence of any commercial or financial relationships that could be construed as a potential conflict of interest.
