# Supplementary material for: Introgressing Subgenome Components from Brassica rapa and B. carinata to B. juncea for Broadening Its Genetic Base and Exploring Intersubgenomic Heterosis
Source: Front Plant Sci. 2016 Nov 17;7:1677. doi: 10.3389/fpls.2016.01677 (PMC5112257; doi:10.3389/fpls.2016.01677)
Supplement: Supplementary file 9 [file Table9.DOCX]

**Supplementary Table 9. Evaluation of the genomic variation and constitution of the new-type *B. juncea* lines at the F_3_ generation**

| Code of F_2:3_ family | Total polymorphic markers | | The ratio of different types of polymorphic markers (%) | | |
| --- | --- | --- | --- | --- | --- |
|  | Mean | Coefficient of variation within the family (%) | A^j^B^j^-specific | Hexaploid-specific | Novel** |
| C34J27-1 (9)* | 215.0 | 5.3% | 28.5 | 31.3 | 40.2 |
| C18J19-1 (19) | 209.4 | 5.3% | 38.1 | 21.4 | 40.4 |
| C28J04-1 (3) | 207.3 | 7.3% | 39.7 | 34.8 | 25.5 |
| C18J02-2 (19) | 207.3 | 6.3% | 41.3 | 24.2 | 34.5 |
| C18J05-2 (8) | 201.1 | 9.8% | 42.1 | 22.6 | 35.2 |
| C18J21-1 (3) | 200.3 | 2.6% | 42.2 | 21.2 | 36.6 |
| C31J35-1(13) | 199.3 | 9.1% | 42.3 | 22.8 | 34.9 |
| Average | 205.7 | 6.52% | 39.2 | 25.5 | 35.4 |

* The number of evaluated plants is shown in parentheses.

** ‘Novel’ means those novel genetic variation including newly appearing alleles and missing alleles in the new-type *B. juncea* lines compared with the parents.
