# Supplementary material for: Introgressing Subgenome Components from Brassica rapa and B. carinata to B. juncea for Broadening Its Genetic Base and Exploring Intersubgenomic Heterosis
Source: Front Plant Sci. 2016 Nov 17;7:1677. doi: 10.3389/fpls.2016.01677 (PMC5112257; doi:10.3389/fpls.2016.01677)
Supplement: Supplementary file 12 [file Image1.PDF]

(A)

(B)

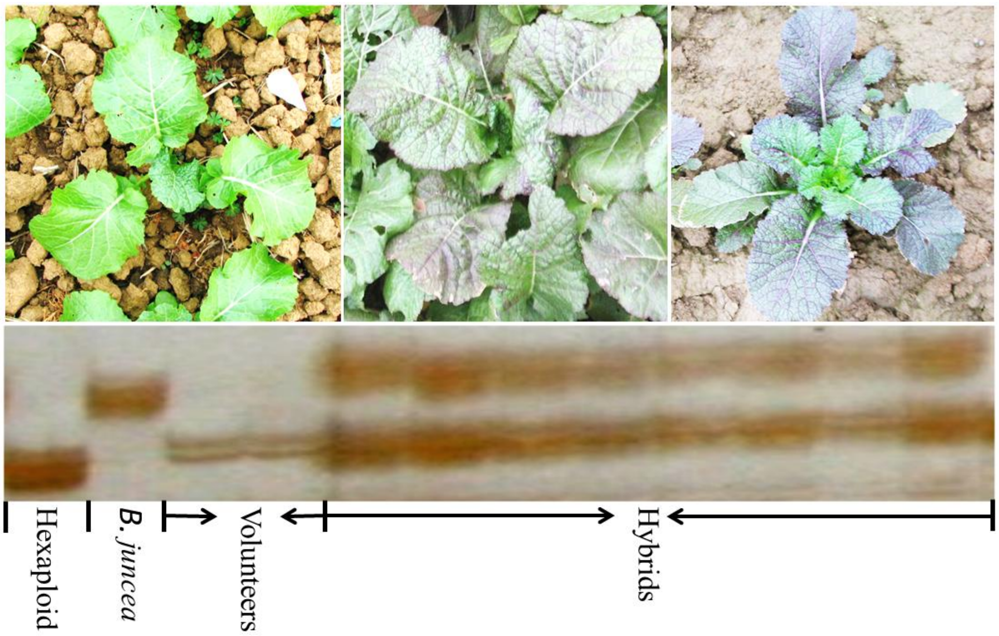

Supplementary Fig. 1. Identification of true pentaploid hybrids based on morphology and molecular markers. (A) The morphology from left to right is hexaploid, hybrid, and traditional *B. juncea*, respectively. (B) An example of identifying true hybrids using molecular markers. Twenty plants from one F_1 cross along with the parents were screened using the SSR primer (Ni4-F08), and two volunteers are presented. The SSR primer Ni4-F08 (forward primer: GAGAAAGAAGCAAACACAAAGC; reverse primer: TCTCTTTCTTCTCGTTGCCG) was used to identify true hybrids from the allohexaploid and traditional *B. juncea*. "Volunteers" means the wild plants generated from the seeds in the soil from last seasons.
