# Supplementary material for: Introgressing Subgenome Components from Brassica rapa and B. carinata to B. juncea for Broadening Its Genetic Base and Exploring Intersubgenomic Heterosis
Source: Front Plant Sci. 2016 Nov 17;7:1677. doi: 10.3389/fpls.2016.01677 (PMC5112257; doi:10.3389/fpls.2016.01677)
Supplement: Supplementary file 13 [file Image2.pdf]

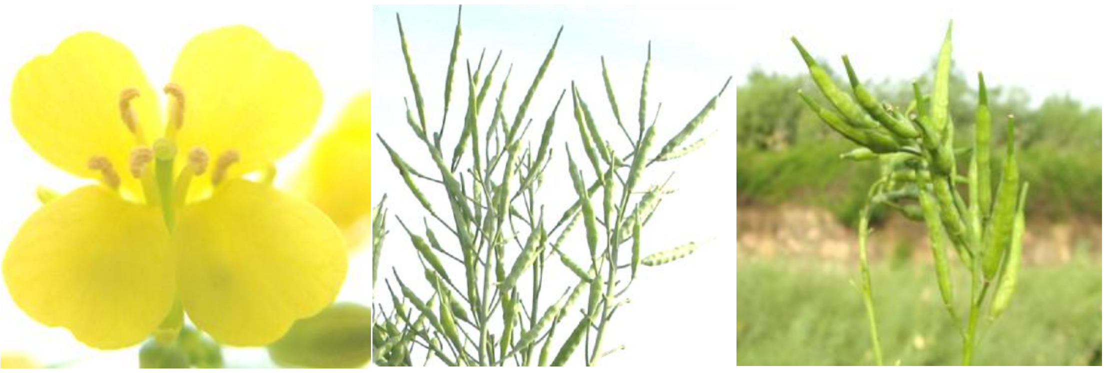

Supplementary Fig. 2. The reproductive organs exhibiting good fertility at the F_3 generation. The three pictures in the left, middle and right shows a flower with abundant pollen at blooming (left), developing siliques after self-pollination (middle) and cross-pollinated with pollen from traditional *B. juncea* (right), respectively.
