# Supplementary material for: Introgressing Subgenome Components from Brassica rapa and B. carinata to B. juncea for Broadening Its Genetic Base and Exploring Intersubgenomic Heterosis
Source: Front Plant Sci. 2016 Nov 17;7:1677. doi: 10.3389/fpls.2016.01677 (PMC5112257; doi:10.3389/fpls.2016.01677)
Supplement: Supplementary file 14 [file Image3.PDF]

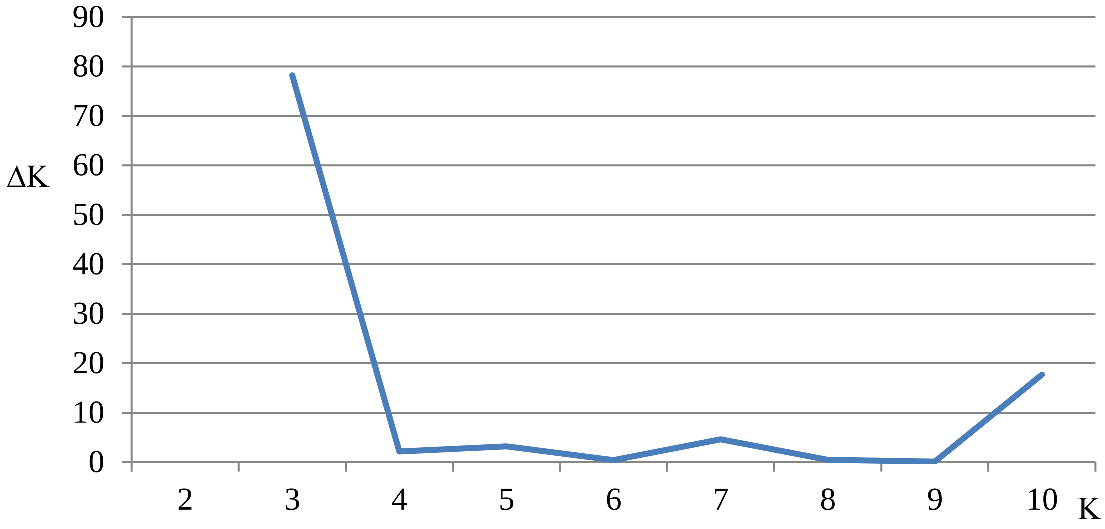

Supplementary Fig. 3. Calculation of the ΔK value for inference of the population genetic structure of the new-type *B. juncea* lines and their parents. The samples contained five lines of hexaploid, eight accessions of traditional *B. juncea* and eight individuals of new-type *B. juncea* in F_3 generation. The markers involved in the study were from A, B and C genome.
