# Supplementary material for: Introgressing Subgenome Components from Brassica rapa and B. carinata to B. juncea for Broadening Its Genetic Base and Exploring Intersubgenomic Heterosis
Source: Front Plant Sci. 2016 Nov 17;7:1677. doi: 10.3389/fpls.2016.01677 (PMC5112257; doi:10.3389/fpls.2016.01677)
Supplement: Supplementary file 15 [file Image4.PDF]

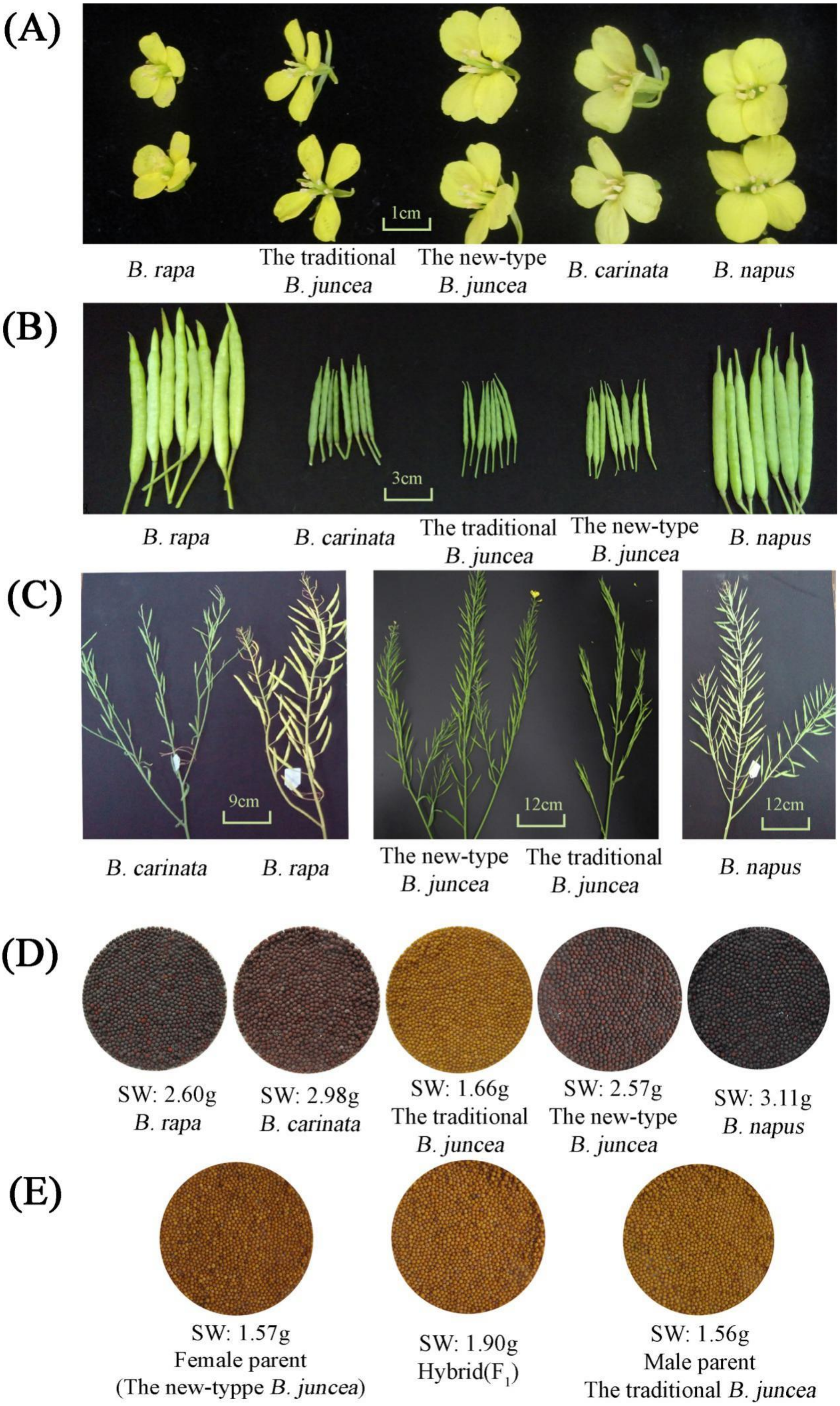
(A)
B. rapa
The traditional B. juncea
The new-type B. juncea
B. carinata
B. napus
1cm
(B)
B. rapa
B. carinata
The traditional B. juncea
The new-type B. juncea
B. napus
3cm
(C)
B. carinata
B. rapa
9cm
The new-type B. juncea
The traditional B. juncea
12cm
B. napus
12cm
(D)
SW: 2.60g
B. rapa
SW: 2.98g
B. carinata
SW: 1.66g
The traditional B. juncea
SW: 2.57g
The new-type B. juncea
SW: 3.11g
B. napus
(E)
SW: 1.57g
Female parent
(The new-typpe B. juncea)
SW: 1.90g
Hybrid(F1)
SW: 1.56g
Male parent
The traditional B. juncea

Supplementary Fig. 4. Demonstration of the improvement of agronomic traits of new-type *B. juncea* lines in the F_6 generation compared with their parental traditional *B. juncea* accessions. (A) The floral morphology of new-type *B. juncea* lines compared with its parental species and *B. napus* cultivars. The lines of new-type *B. juncea* shows nearly similar flower size as *B. napus* cultivars, and obvious bigger size of flower than its parental *B. juncea* accession. (B) The silique of new-type *B. juncea* lines and the comparison with the accession of its parental species and *B. napus*. New-type *B. juncea* shows increased silique length than traditional *B. juncea*, but is still smaller than that of *B. napus*. (C) The branches and the silique lift degree of new-type *B. juncea* lines and the comparison with the accession of its parental species and *B. napus*. The silique lift is obviously improved compared to traditional *B. juncea*. (D) The thousand seed weight of new-type *B. juncea* lines and the comparison with the accession of its parental species and *B. napus*. The seed weight of new-type *B. juncea* is not as good as *B. napus*, but shows obvious improvement compared to traditional *B. juncea*. (E) The strong heterosis on the trait of thousand seed weight of the intersubgenomic hybrids between new-type *B. juncea* lines and traditional *B. juncea* accessions.
